# Guillain‐Barré syndrome after mRNA‐1273 (Moderna) COVID‐19 vaccination: A case report

**DOI:** 10.1002/ccr3.5733

**Published:** 2022-04-14

**Authors:** Zauraiz Anjum, Charoo Iyer, Sidra Naz, Vikash Jaiswal, Gaurav Nepal, Maryrose Laguio‐vila, Sanjay Anandaram, Sahil Thapaliya

**Affiliations:** ^1^ 6932 Rochester General Hospital Rochester New York USA; ^2^ 1811 Harvard Medical School Boston Massachusetts USA; ^3^ AMA school of medicine Makati Philippines; ^4^ 113015 Tribhuvan University Institute of Medicine Kathmandu Nepal

**Keywords:** COVID‐19, GBS, Moderna, mRNA, vaccine

## Abstract

Guillain‐Barre syndrome (GBS) is an acquired inflammatory polyradiculoneuropathy that often follows infection with a virus or bacteria and in rare occasions, vaccination may precede GBS. We present a case of 80‐year‐old male patient who presented with chief complaints of progressive, ascending bilateral lower extremity paresthesia and weakness following first dose of Moderna vaccine. His symptoms got exacerbated after 2nd dose. Clinical examination and investigation findings including lumbar puncture, nerve conduction study, and electromyography were consistent with the diagnosis of GBS. The patient received treatment with intravenous immunoglobulin and there was significant improvement toward the end of 5th day. Though rare, this case report suggest that physician should remain vigilant for GBS following COVID‐19 vaccination.

## Graphical abstract

KCM: Our case highlight the need for vigilance in patients with neurologic symptoms after COVID‐19 vaccination and for postvaccination surveillance programs to assess causality of GBS.

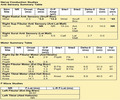

## 1 INTRODUCTION

Guillain‐Barre Syndrome is an acquired inflammatory polyradiculoneuropathy that often emerges as a result of recent infections. Infection with *Campylobacter jejuni*, *Mycoplasma pneumoniae*, Cytomegalovirus, Zika virus, Influenza virus, and influenza vaccine injection are all common GBS triggers.^1^, ^2^ GBS has also been reported during or after COVID‐19 infection, caused by severe acute respiratory syndrome coronavirus 2 (SARS‐CoV‐2).^3^ A worldwide mass vaccination operation has been underway to combat COVID‐19 since the beginning of 2021. COVID‐19 vaccine‐related GBS is becoming more common as the COVID‐19 vaccination program spreads around the world. GBS has been linked to both messenger‐RNA and adenovirus‐vectored COVID‐19 vaccines.^4^, ^5^ Here, we describe a case of GBS developing after receiving the first dose of mRNA‐1273 (Moderna COVID‐19 vaccine). In this case, the temporal linkage between mRNA‐1273 immunization and GBS development suggested a vaccine‐induced etiology, and the clinical consequences of this link merit further investigation.

## 2 CASE PRESENTATION

An 80‐year‐old male patient presented to emergency room with chief complaints of progressive, ascending bilateral lower extremity paresthesia, and weakness following COVID‐19 (Moderna) vaccine. He received his first dose of Moderna vaccine in the last week of January 2021, following which he noticed bilateral lower extremity paresthesia (tingling and numbness), which was followed by progressive bilateral lower limb distal weakness, beginning at his feet and then ascending to involve his legs, knees, and hips. He was unable to maintain balance and started having multiple falls prompting him to start using a walker for ambulation. He then received the second dose of his vaccine in February 2021. The next morning, he was unable to get out of his bed due to severe bilateral lower extremity weakness, and he therefore presented to the emergency room.

The patient's past medical history was notable for COVID‐19 infection the previous year, from which he had an uneventful recovery, chronic lymphocytic leukemia (CLL) without the end organ involvement for which he had not received any treatment, degenerative disease of lumbar spine, remote history of TIA, hypertension, and hyperlipidemia. The patient denied recent trauma, exposure to drugs / toxins, recent infection, auto‐immune disease, family history of hereditary neuropathy or personal history of smoking, alcohol, and drug dependence.

On examination, vitals were notable for mild hypertension, temperature of 101.4 F. Neurological examination was notable for intact power and sensations in bilateral upper limbs, normal bilateral brachioradialis, and triceps and biceps reflexes. In lower extremity, power was 2/5 at all ankle, knee, and hip joints. Bilateral ankle reflexes were absent, and patellar reflexes were markedly reduced left knee, absent in right knee. Sensation to light touch was decreased from knee downwards. Proprioception diminished in bilateral toes. Higher mental function and cranial nerves were intact. General, cardiovascular, respiratory, and abdominal examination were otherwise normal.

**FIGURE 1:**
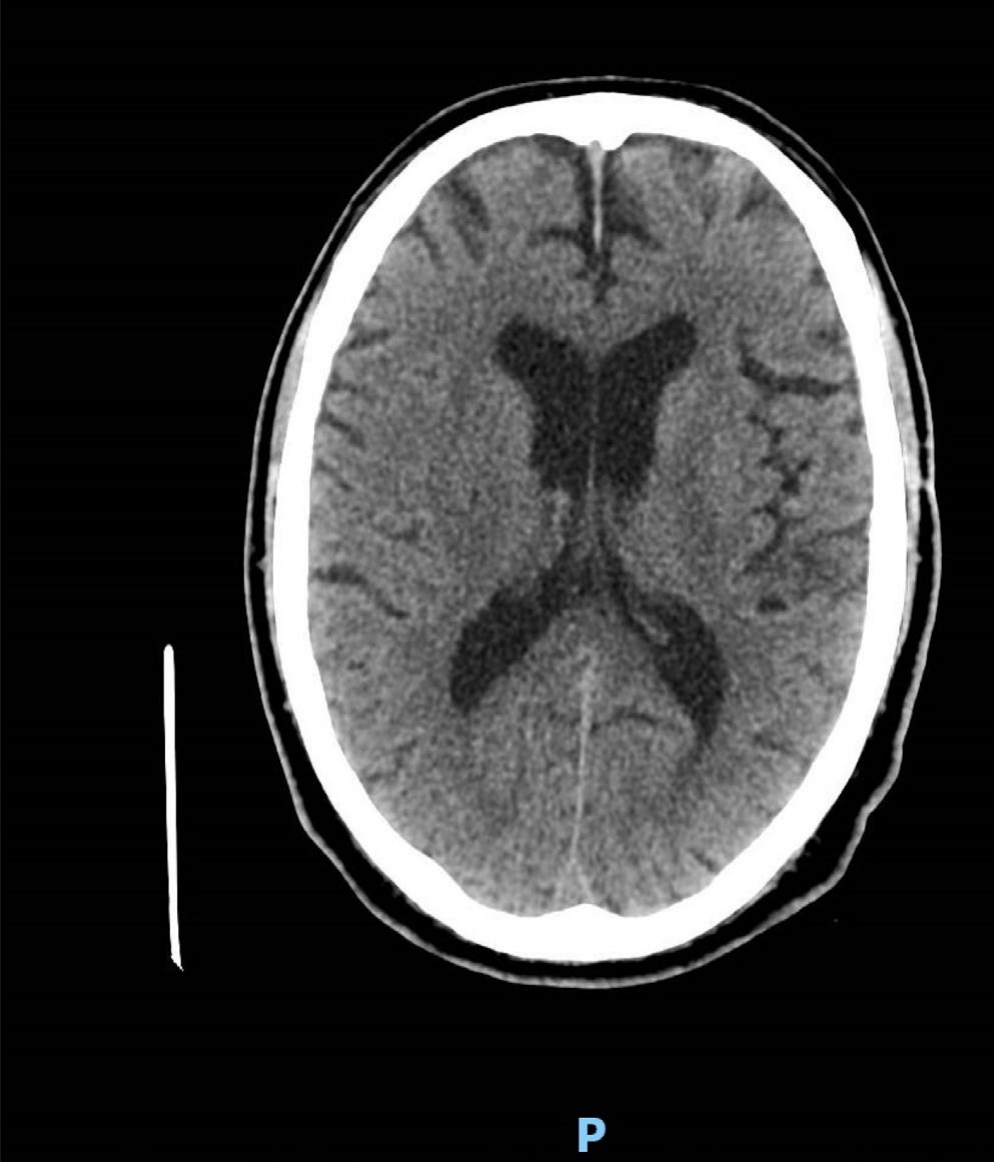
Head computed tomography showing normal scan

**FIGURE 2:**
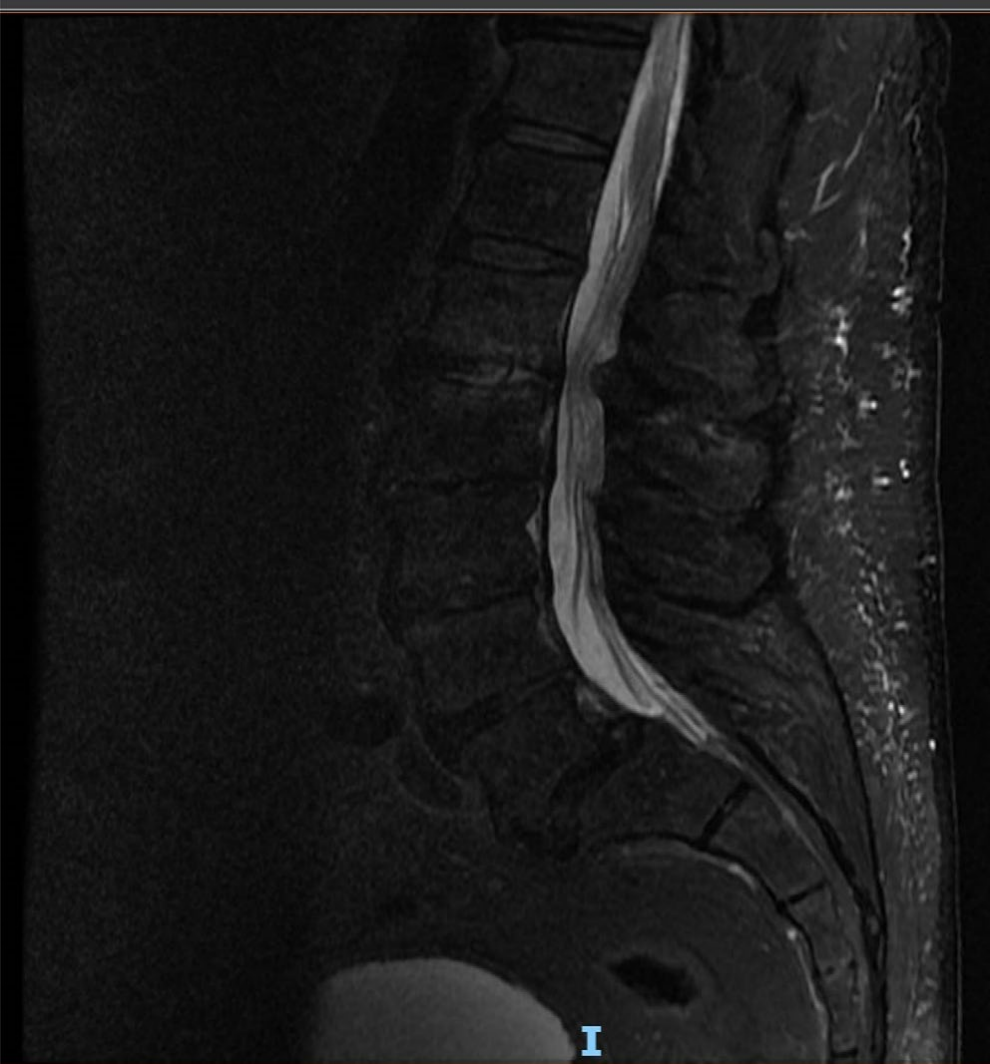
Magnetic resonance imaging of lumbar spine showing degenerative changes

Laboratory findings were notable for lymphocyte predominant leukocytosis (in the setting of CLL). Computed tomography (CT) head was negative for any mass occupying lesion or infarct or hemorrhage (Figure 1). Magnetic resonance imaging (MRI) of lumbar spine without IV contrast showed findings consistent with degenerative disease (Figure 2). Infectious work‐up including blood culture and urine analysis was negative. Lumbar puncture was done, which showed colorless cerebrospinal fluid (CSF) with albuminocytological dissociation.

**FIGURE 3:**
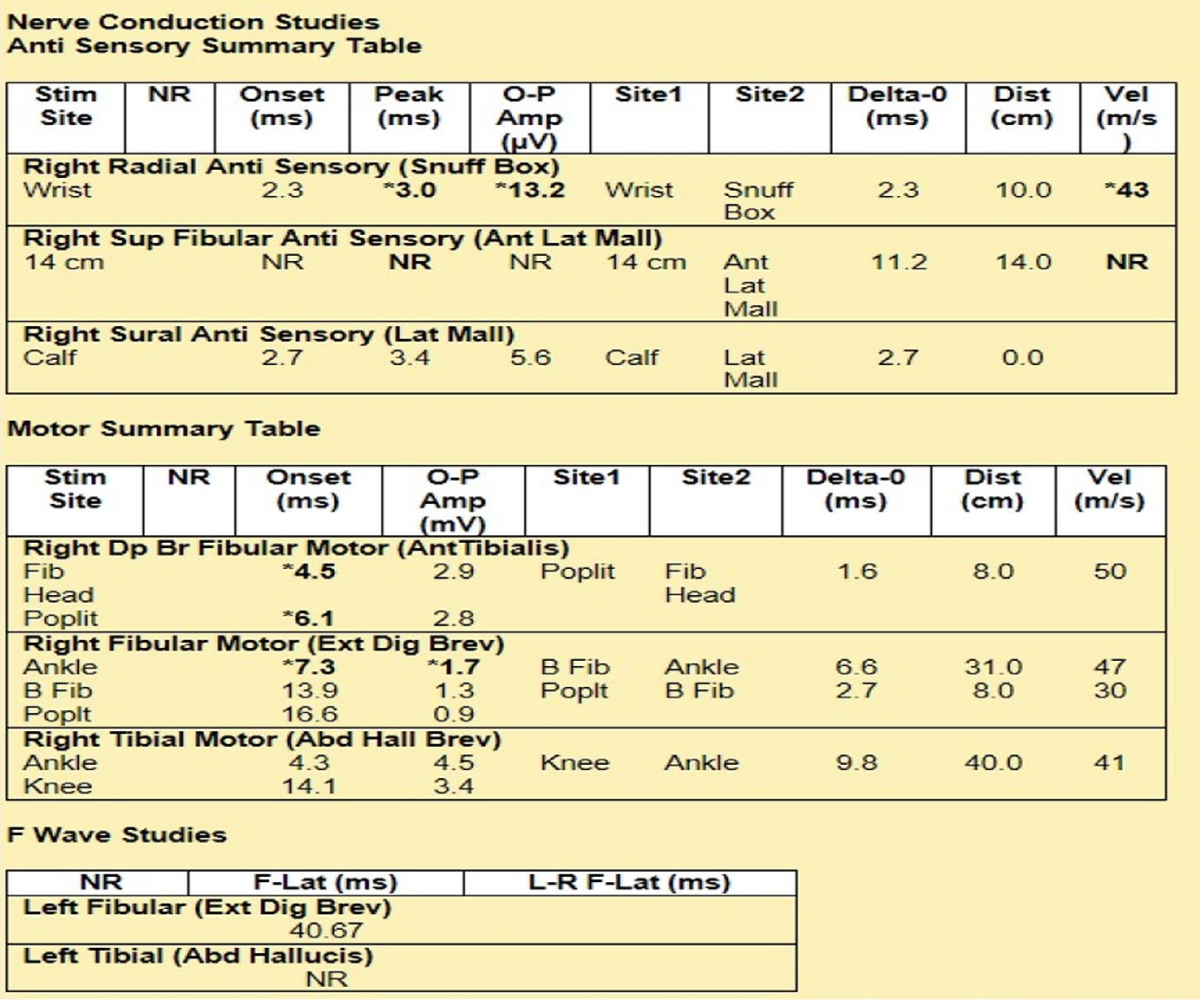
Nerve conduction studies of both sensory and motor nerves

**FIGURE 4:**
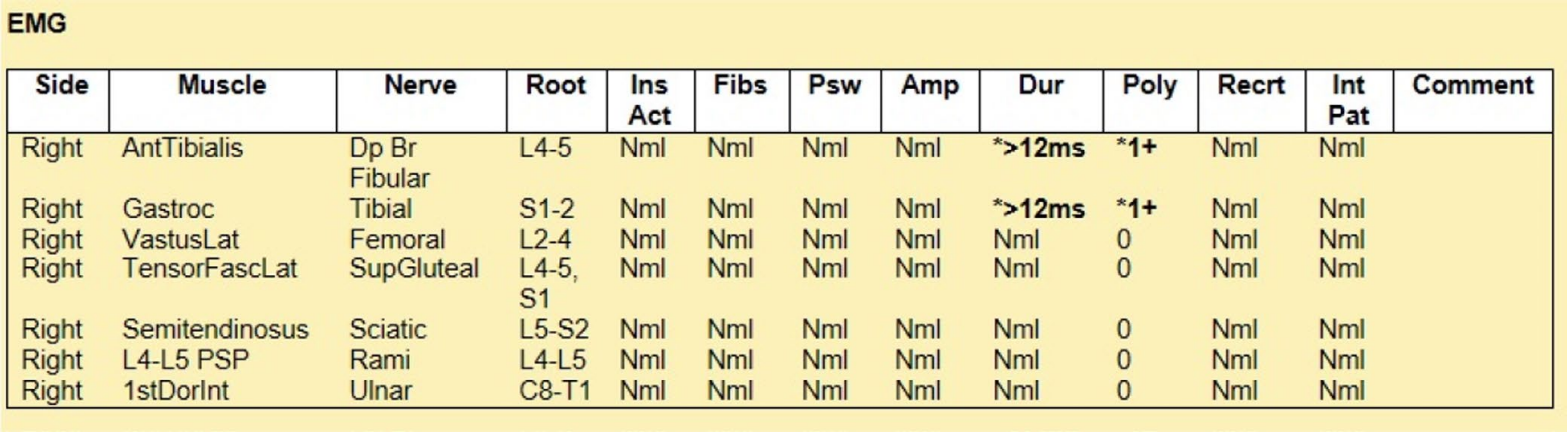
Electromyography studies of selected muscles

The patient's CSF findings of albumin‐cytological dissociation along with his classic presentation of rapidly progressive ascending bilateral lower limb weakness, with diminished deep tendon reflexes and sensory loss were concerning for acute inflammatory demyelinating polyneuropathy (AIDP). Accordingly, patient was started on intravenous immunoglobulin (IVIG) at 400 mg/kg/day for 5 days. Patient tolerated this well without any side effects or laboratory abnormalities. Daily negative inspiratory force (NIF) test was done and remained negative throughout. Toward the end of his 5 days of IVIG therapy, the patient noted significant improvement in bilateral lower extremity strength, and began to ambulate with help from physical therapy. Power in hip flexors was +2 on the right and improved to +3 on the left, though he still had some residual dorsiflexion and plantar flexion weakness in the right foot. The patient was discharged to rehabilitation facility. He underwent outpatient nerve conduction study (NCS) (Figure 3) and electromyography (EMG) (Figure 4), which showed widespread demyelinating polyneuropathy affecting the lower extremities more than the upper extremities, consistent with AIDP. There was an evidence of renervation without ongoing denervation on EMG which often indicates that AIDP is in the recovery stage. The patient has been making slow but steady recovery in terms of his strength and ambulation since then.

## 3 DISCUSSION

Guillain‐Barré syndrome (GBS) is a rare, but potentially fatal, immune‐mediated disease of the peripheral nerves and nerve roots that is usually triggered by infections. The pathogenesis involves molecular mimicry, with the immune response elicited by an antecedent trigger (infection or vaccine) cross‐reacting with the structural components of peripheral nerves.^1^, ^5^ The resulting demyelination characteristically presents as symmetric muscle weakness and depressed or absent deep tendon reflexes that progresses over a period of a couple of weeks. Diagnosis is suspected based on the clinical features, with albuminocytological dissociation seen on CSF analysis providing additional supportive evidence. Confirmation and further stratification into various subtypes can be done with the help of electrodiagnostic studies (EMG and NCS) and CSF antibody tests.^2^, ^6^

The most common and well‐established trigger for GBS is *C.* *jejuni* infection.^7^, ^8^ GBS has also been associated with viral infections, notably human immuno deficiency virus, Epstein Barre virus, influenza virus, cytomegalo virus, and Zika virus, though a clear causal relationship has not been established.^1^ Although infection is the common trigger, a small percentage of patients have been noted to develop GBS following other inciting events, including immunization.^6^ The vaccine that has been most discussed in this regard is the influenza vaccine, with reports of its association with GBS emerging after a 1976 vaccination program in New Jersey^9^ since then multiple studies have reported an association surrounding this.^10^, ^11^, ^12^ Cases of GBS have also been reported after recombinant Zoster, quadrivalent meningococcal, and tetanus toxoid vaccinations.^1^, ^13^

The ongoing COVID‐19 pandemic has raised the concern of possible association between COVID‐19 infection and GBS.^14^, ^15^ The subsequent COVID‐19 vaccination drives and the VAERS (Vaccine Adverse Event Reporting System) have also led to questions regarding COVID‐19 vaccine being a potential trigger for GBS.^16^ From immunization standpoint, in the United States, two mRNA vaccines the BNT162b2 (Pfizer‐BioNTech COVID‐19 vaccine) and the COVID‐19 mRNA vaccine mRNA‐1273 (Moderna COVID‐19 vaccine) as well as the adenoviral vector vaccine Ad26.COV2.S (Janssen or the Johnson & Johnson vaccine) are in use for the prevention of COVID‐19. The adeno virus vaccine ChAdOx1 nCoV‐19/AZD1222 (Astrazeneca) is being used in the United Kingdom, Canada, and India.^17^

There are multiple cases of GBS being reported following the adenovirus vaccine administration.^18^ As of July 24, 2021, 130 reports of presumptive GBS were identified in VAERS following Ad26.COV2.S vaccination.^19^ In Europe until June 2021, around 227 cases of GBS have been noted after administration, of around 51 million doses of the AstraZeneca vaccine.^20^ There does not seem to be the same degree of association between the mRNA vaccine and the development of GBS, though a couple of cases have been reported.^18^

Our patient did have antecedent history of COVID‐19 infection; however, he made a fairly uneventful recovery from this and it was nearly 3 months prior to his presentation with GBS. He started noticing bilateral lower extremity weakness only shortly after his first dose of Moderna Vaccine, which progressed acutely after the second dose, showing a clear temporal association. Other causes were excluded based on history, examination, and radiological/laboratory investigations as noted. The CSF findings of albuminocytological dissociation and the clinical response to IVIG therapy further supports the diagnosis of GBS while the EMG/NCS findings confirm it. This is one of the few reported case of AIDP following Moderna vaccine administration. This case does serves as important reminder to be on the lookout for similar cases and to consider COVID‐19 vaccination as a possible differential in GBS cases presenting to various clinical settings.

## 4 CONCLUSION

We describe a case of GBS following the administration of mRNA‐1273 (Moderna) vaccine against COVID‐19. Though the association between mRNA vaccine and development of GBS is yet to be determined, GBS should be considered as a possible differential on the lookout for similar cases following vaccination.

## CONFLICT OF INTEREST

The authors declare that they have no competing interests.

## AUTHOR CONTRIBUTIONS

ZA, CL, ML, and SA were involved in patient care (diagnosis, treatment, and follow‐up). ZA, GN, CL, SN, VJ, ML, and SA contributed to the collection of case information, writing of the manuscript, and manuscript revision. ZA, GN, CL, and ST were involved in revising the manuscript critically for important intellectual content. All authors approved the final version.

## ETHICAL APPROVAL

This study did not include experiments on animals or humans. The patient gave consent to use his details for this case study.

## CONSENT

Written informed consent was obtained from the patient for publication of this case report and any accompanying images. A copy of the written consent is available for review by the Editor‐in‐Chief of this journal.

## Data Availability

The data used in the case report are available on reasonable request.
